# Supplementary material for: Curated and harmonised transcriptomics datasets of interstitial lung diseases
Source: Data Brief. 2025 Oct 14;63:112139. doi: 10.1016/j.dib.2025.112139 (PMC12581653; doi:10.1016/j.dib.2025.112139)
Supplement: Supplementary file 1 [file mmc1.zip › Supplementary_material/RNA-seq/fastQC reports/GSE184316/SRR15926776_fastqc.html]

SRR15926776.fastq.gz FastQC Report 

FastQC Report

Mon 5 Feb 2024  
SRR15926776.fastq.gz

## Summary

- Basic Statistics
- Per base sequence quality
- Per sequence quality scores
- Per base sequence content
- Per sequence GC content
- Per base N content
- Sequence Length Distribution
- Sequence Duplication Levels
- Overrepresented sequences
- Adapter Content

## Basic Statistics

| Measure | Value |
| --- | --- |
| Filename | SRR15926776.fastq.gz |
| File type | Conventional base calls |
| Encoding | Sanger / Illumina 1.9 |
| Total Sequences | 5882999 |
| Sequences flagged as poor quality | 0 |
| Sequence length | 25-372 |
| %GC | 51 |

## Per base sequence quality

## Per sequence quality scores

## Per base sequence content

## Per sequence GC content

## Per base N content

## Sequence Length Distribution

## Sequence Duplication Levels

## Overrepresented sequences

| Sequence | Count | Percentage | Possible Source |
| --- | --- | --- | --- |
| CACCTCCCCGAAGCTCAGGGAGAGCCCTGTTAGGGCCGCCTCTGGCCCTA | 98404 | 1.67268428908453 | No Hit |
| CAGTGGGGCCACGAGCTGAGTGCGTCCTGTCACTCCACTCCCATGTCCCT | 88447 | 1.503433877857195 | No Hit |
| AGCTGCTAAGTGCTGTGTTGTCGTTCCCCCTGCTTAAAATAAAGTTGTTT | 44054 | 0.7488357553689878 | No Hit |
| CCCTGGCTGCTACAGGAGAATAGCAGACAGGTATAGTTAAGAAACAACTT | 42705 | 0.7259052738237759 | No Hit |
| CAGCAGTTTACACCTGCTAGGGTGTTCAAAGGTCAGTGCTATAGAAATTC | 41865 | 0.7116268420239406 | No Hit |
| AACAAGCACAAAGCCAAGAAAACCAACGATGCCAGATACTGAATTTCTAT | 33266 | 0.5654598955396729 | No Hit |
| AGACCACCAGCAGCCTGCCCAGGGCCTCACCACCAACTTC | 29574 | 0.5027027881527771 | No Hit |
| GCCGACAAGACCAACGTCAAGGCCGCCTGGGGTAAGGTCGGCGCGCACGC | 29133 | 0.49520661145786354 | No Hit |
| AGGTCTTGGTGGTGGGGAAGGACAGGAACATCCTCTCCAGGGCCTCCGCA | 27079 | 0.46029244608064696 | No Hit |
| GAAGTTGGTGGTGAGGCCCTGGGCAGGCTGCTGGTGGTCT | 24255 | 0.41228971822024785 | No Hit |
| AGAGGGCTCCCAGAAGTGTGTGGCTGAGCTGGGTCCCCAGGCCGTGGGGG | 22244 | 0.37810647256611807 | No Hit |
| CCAGTCTCGGCTCAGCCAAACACTGTCAGGGCCCCCAGCAGGGCCTTCAG | 22196 | 0.377290562177556 | No Hit |
| TGATATGGTTCCTGGCAAGCCCATGTGTGTTGAGAGCTTCTCAGACTATC | 18987 | 0.32274355307556574 | No Hit |
| CAATCCACTTGATCCCAACTCATCTCTCATTTATTTCGGCTTCTTTTATT | 18907 | 0.3213837024279623 | No Hit |
| GAGTGCGCTGCACTGACCTTCTTCCAAGCCTCAGTTCCTGTTCTAGGAAC | 18810 | 0.31973488351774326 | No Hit |
| AGGCTACACACTGAGGACTCTGTTCCTCCCCTTTCCGCCTAGGGGAAAGT | 18386 | 0.3125276750854453 | No Hit |
| GCCAACACACAAGCAGTTCACAGTAGAACACTGCAGACTGCAGGGCATTT | 17716 | 0.3011389259117671 | No Hit |
| TGATGACACCCACCGCAACTGTCTGTCTCATATCACGAACAGCAAAGCGA | 17672 | 0.3003910080555853 | No Hit |
| GGAAGACAGTGTGGCGATTCCTCAGGGATCTAGAACTAGAAATACCATTG | 17584 | 0.29889517234322155 | No Hit |
| TTCAGCCCTGATCAAGACATGAGGGAGGCAGGGGCTCAGCTGAAGAAGCT | 17145 | 0.29143299191449806 | No Hit |
| GCAGCTGCTTCCGCATAGCTGCTGTGGTCAAAAAGGAGCCCAGAGTGACA | 16744 | 0.2846167405433861 | No Hit |
| TGGGTCTGCGGTGACCCTATGCATTCCTTCAGTGCTTGCTAGAACAGTTT | 16693 | 0.28374983575553897 | No Hit |
| TCTTGGGTTATAATCATTGGCAATGTTGTAATCAACAACCCATGTTATTT | 16105 | 0.27375493349565416 | No Hit |
| ATTGATTCTTCCTACCCATGAGCATGGAATGTTCTTCCATTTGTTTGTAT | 15053 | 0.2558728974796698 | No Hit |
| ACAACAAACAGAACGGCGACCGTCAAGGAAAACTGTCACTCTGGGCTCCT | 14551 | 0.24733983466595863 | No Hit |
| GAAGGACCTCTTCAAGGAGAACTACAAACCACTGCTCAAGGAAATAAAAG | 14302 | 0.24310729952529314 | No Hit |
| GGATGGAGCAGGGCAAGGCCTCAAACCGTTTCAAAACTGTTCTAGCAAGC | 13836 | 0.2351861695030035 | No Hit |
| TTACAAAAGCAGAATAAAAGCGAAAAGAAATGAAAATGTTACACTACATT | 13481 | 0.22915183225426353 | No Hit |
| GGAAAGGGGGCACTGACTGGTGATGCTGATATTGGAGTGCATCTGAGGCT | 12886 | 0.21903794306271343 | No Hit |
| GGGAAGCGGGGAATGTCTACGTGCGTATGCACGTGGCACTCTCTGCCCGA | 12691 | 0.2157233071091802 | No Hit |
| GACGAGTCAGAAAGGTCACGTCCTGCTCTTGGTCCTTGTCAGTGCCATGT | 11790 | 0.2004079891905472 | No Hit |
| CACTGTTTCTTATAGCGGTGGTTTAAACAGAGGTGCAAACAGCAAGCGGA | 11372 | 0.19330276955681958 | No Hit |
| ATAGCAGCATGATTTATAGTCCTTTGGGTATATACCCAGTAATGGGATGG | 10575 | 0.17975525748007096 | No Hit |
| CAATAAATAGGACACTTCTGCCAAAGGAACTCGTGCACAGCACCACAGAA | 10538 | 0.1791263265555544 | No Hit |
| AGGCCCCAAAAGAAAACAGCGTCTTACTGGGGAGGGATATGTGCTTTCTC | 10365 | 0.17618564953011212 | No Hit |
| TTTTTCCATGAGCTTAATGATGCTTTCTCTGGGCTTTTGGGGGAGGGTGT | 9788 | 0.16637772673427279 | No Hit |
| GAAGGACCTCTTCAAGGAGAACTACAAACCACTGCTCAATGAAATAAAAG | 9624 | 0.16359003290668586 | No Hit |
| CTTCATGATGTAGCAGCAGGTGCCAGGGGCTGGCTTGTAGGCGATCAGCA | 9067 | 0.15412207277274736 | No Hit |
| TACCACTGCCACCTTCTCCATCGGCTCCACTGGCCTCGTGGTGTATGACT | 8697 | 0.14783276352758176 | No Hit |
| GGCAATGAGCGGTTCCGCTGCCCTGAGGCACTCTTCCAGCCTTCCTTCCT | 8576 | 0.14577598942308168 | No Hit |
| CCAGGGACTTTTTGTTGCCTGTAAAGTGCTCTGGCATTGCCTGAGGATAG | 8089 | 0.13749789860579612 | No Hit |
| GTCCACGTCACACTTCATGATGGAGTTGAAGGTAGTTTCGTGGATGCCAC | 8089 | 0.13749789860579612 | No Hit |
| GGCGCAGGGGTACGGACCTCAGCTGAGTCATGGGAGCTGAATGTATGTGT | 8064 | 0.13707294527842007 | No Hit |
| TTTTCCATGAGCTTAATGATGCTTTCTCTGGGCTTTTGGGGGAGGGTGTC | 7855 | 0.13352033546155626 | No Hit |
| CAATGGGGTACTTCAGGGTCAGGATGCCACGCTTGCTCTGGGCCTCGTCG | 7152 | 0.12157064789574161 | No Hit |
| CAAGGAAAGAGAGGCCACAGTAAGGCCCATCCGAGGTCCTGAGGAGATCT | 7054 | 0.11990483085242748 | No Hit |
| AGTTGCACTTGGCTTCACCCGTGTGACTTTCGTAACGGGGAGAGAGAGAA | 7022 | 0.11936089059338612 | No Hit |
| TGAGGGGCACGGCCACAGCCCCCCAAAGGCGACAAGATCCGCTTGCTGTT | 7021 | 0.11934389246029109 | No Hit |
| GTCTCAACATCTGTGTTGGGGAGAGTGGAGACAGACTGACGCGAGCAGCC | 6370 | 0.10827810781541863 | No Hit |
| CAGCCGTGGCAGTCTCCCTTCCTAGCCATGGAAGAGCATATCCTTGTTTA | 6248 | 0.1062043355778235 | No Hit |
| AGACCACCAGCAGCCTGCCCAGGGCCTCACCACCAACTTCATCCACGTTC | 6160 | 0.10470849986545978 | No Hit |
| GTGTCCAGAGACAACACTAGACCAGTACTATGATCCCAAGTTACTCTTTG | 5974 | 0.10154684710978193 | No Hit |

## Adapter Content

Produced by FastQC (version 0.11.7)
